# In Vivo Evaluation of Safety and Efficacy of Ethyl Cellulose-Ethanol Tissue Ablation in a Swine Cervix Model

**DOI:** 10.3390/bioengineering10111246

**Published:** 2023-10-25

**Authors:** Tri T. Quang, Jeffrey Yang, Michele L. Kaluzienski, Anna Parrish, Asma Farooqui, David Katz, Brian Crouch, Nimmi Ramanujam, Jenna L. Mueller

**Affiliations:** 1 Fischell Department of Bioengineering, University of Maryland, College Park, MD 20742, USA; tritquang@gmail.com (T.T.Q.); jyang12@umd.edu (J.Y.); mkaluzi4@umd.edu (M.L.K.); annagparrish@gmail.com (A.P.); asmafarooqui101@gmail.com (A.F.); 2 Center for Interventional Oncology, Radiology and Imaging Sciences, NIH Clinical Center, National Cancer Institute, National Institutes of Health, Bethesda, MD 20892, USA; 3 Department of Biomedical Engineering, Duke University, Durham, NC 27708, USA; dkatz@duke.edu (D.K.); brian.crouch@duke.edu (B.C.); nimmi.ramanujam@duke.edu (N.R.); 4 Department of Obstetrics and Gynecology, Duke University School of Medicine, Durham, NC 27710, USA; 5 Duke Global Health Institute, Duke University, Durham, NC 27710, USA; 6 Department of Pharmacology and Cancer Biology, Duke University, Durham, NC 27708, USA; 7 Department of OB-GYN & Reproductive Science, University of Maryland School of Medicine, Baltimore, MD 21201, USA; 8 Marlene and Stewart Greenebaum Cancer Center, University of Maryland School of Medicine, Baltimore, MD 21201, USA

**Keywords:** ethanol ablation, ethyl cellulose, swine cervix, thermocoagulation, cervical dysplasia, low- and middle-income countries

## Abstract

Current therapies for treating cervical dysplasia are often inaccessible in low and middle-income countries (LMICs), highlighting the need for novel low-cost therapies that can be delivered at the point of care. Ethanol ablation is a low-cost therapy designed to treat locoregional cancers, which we augmented into an ethyl cellulose (EC)-ethanol gel formulation to enhance its efficacy. Here, we evaluated whether EC-ethanol ablation is able to safely achieve an ablation zone comparable to thermocoagulation, a commonly used therapy for cervical dysplasia. The study was performed in 20 female Yorkshire pigs treated with either a single 500 µL injection of EC-ethanol into the 12 o’clock position of the cervix or a single application of thermocoagulation at 100 °C for 20 s. The average temperature, heart rate, respiratory rate, and blood oxygen remained within normal ranges throughout the EC-ethanol procedure and were similar to the thermocoagulation group. No major side effects were observed. The reproductive tracts were excised after 24 h to examine ablation zones. Comparable depths of necrosis were seen for EC-ethanol (18.6 ± 1.6 mm) and thermocoagulation (19.7 ± 4.1 mm). The volumes of necrosis induced by a single injection of EC-ethanol (626.2 ± 122.8 µL) were comparable to the necrotic volumes induced by thermocoagulation in the top half of the cervices (664.6 ± 168.5 µL). This suggests that two EC-ethanol injections could be performed (e.g., at the 12 and 6 o’clock positions) to achieve comparable total necrotic volumes to thermocoagulation and safely and effectively treat women with cervical dysplasia in LMICs. This is the first study to systematically evaluate EC-ethanol ablation in a large animal model and compare its safety and efficacy to thermocoagulation, a commonly used ablative therapy for cervical dysplasia.

## 1. Introduction

Cervical cancer is a substantial global burden, with an estimate of 604,127 new cases and 341,831 deaths worldwide in 2020, of which 532,239 new cases (~88.1%) and 312,373 deaths (~91.4%) occurred in low- and middle-income countries (LMICs) [1]. Early-diagnosed lesions, such as cervical dysplasia, can be surgically excised by loop electro-excision procedure (LEEP) or cold knife conization, or they can be locally destroyed by laser vaporization or ablative therapies such as cryotherapy and thermocoagulation [2]. LEEP, cold knife conization, and laser vaporization are usually limited to referral centers in LMICs due to the requirement of specialized equipment and medical expertise [3]. Cryotherapy and thermocoagulation have been endorsed by the World Health Organization to treat cervical dysplasia in LMICs and have become the preferred methods due to their lower risk of periprocedural infection, simplicity, and affordability [4,5]. Cryotherapy is more affordable than excisional procedures; however, it uses high-quality gas tanks connected to a metal probe to freeze lesions. Consequently, cryotherapy can be logistically and economically difficult to implement in population-based programs in LMICs [5,6,7,8,9,10]. Thermocoagulation, in which heat is used to ablate dysplasia, has obtained similar efficacy to cryotherapy, with the added benefits of lower total cost, shorter treatment duration, fewer side effects, and improved patient satisfaction and experience [5,6,7,8,9,10]. However, protocols for temperature, duration, and cycle times are not standardized [10,11]. For example, one study found that the average depth of necrosis after one cycle of thermocoagulation ranged from 2.6 mm (100 °C for 20 s) to 3.5 mm (120 °C for 30 s) [10,11]. Longer heating times at higher temperatures or multiple cycles increase necrotic depth; however, this may increase patient pain and discomfort [12]. High-grade dysplasia can vary in depth from 1.4 to 4.8 mm, and early cancers (i.e., Stage 1A) can extend 3–5 mm deeper into the stroma. Taken together, there is an opportunity to develop alternative efficacious, accessible ablative therapies to treat high-grade cervical dysplasia and early cancers in LMICs.

Ethanol ablation, which involves injecting ethanol into lesions to cause necrosis, is an alternative, low-cost, accessible therapy that only requires medical-grade ethanol, a syringe, and a needle and can be delivered at a variety of depths by changing the depth of needle insertion. Percutaneous ethanol injection (PEI) is a well-established chemical ablative therapy for hepatocellular carcinoma [13,14] and has also been applied for the treatment of pancreatic tumors [15], gastrointestinal stromal tumors [16], and thyroid and parathyroid tumors [17,18]. PEI is typically used to treat tumors adjacent to large blood vessels or critical organs in which thermal ablation is infeasible or unavailable. However, injected ethanol has the propensity to leak away from the injection site into adjacent tissues, particularly in non-encapsulated tumors. Ethanol leakage reduces efficacy at the injection site, consequently requiring additional sessions or injection of larger volumes of ethanol; it also impacts safety as leakage can cause collateral damage and complications due to accumulated ethanol in adjacent structures and other organs [19,20].

To address these challenges, we developed a novel ethyl cellulose (EC)-ethanol formulation that forms a gel-like material upon injection into tissue, sequestering ethanol at the injection location and thereby reducing leakage [19]. We conducted an initial in vivo study in a hamster cheek pouch model of oral squamous cell carcinoma and found that tumor volume was significantly lower for injections of 3% EC-ethanol vs. ethanol alone 7 days after ablation. Reducing the injection rate from 100 mL/h to 10 mL/h further reduced leakage from the tumor [19]. In a second in vivo study in a murine breast cancer model, we found that injection of 6% EC-ethanol significantly increased tumor necrotic volume by 5× and overall survival compared to ethanol alone [21]. In our most recent in vivo application in a rat liver model, we found that 12% EC-ethanol yielded the largest ablation zone compared to other EC concentrations, which was 6× greater than that for pure ethanol [22,23].

In the present study, we scaled up to swine cervices, which are comparable in size to human cervices [24]. We demonstrated that EC-ethanol ablation was able to safely achieve an ablation zone comparable to thermocoagulation in swine cervices (n = 10 per treatment arm). To enable this comparison, we selected a shallow needle insertion depth for EC-ethanol; however, in the future, EC-ethanol could be delivered at deeper depths to treat early cancers. To inject the cervix, we used a custom hand-held injector, which was previously developed and validated in a series of benchtop and preliminary ex vivo experiments [25]. The study here is the first to systematically evaluate EC-ethanol in a large animal model and to compare its performance to a clinically accepted therapy. This is a critical step in translating EC-ethanol ablative therapy to women with cervical dysplasia in LMICs.

## 2. Materials and Methods

### 2.1. Cohort of Swine

The animal study was approved by the University of Maryland Institutional Animal Care and Use Committee (protocol number R-AUG-20-47), and all procedures followed ethical guidelines. Female Yorkshire Cross pigs (n = 20: 10 for EC-ethanol ablation, 10 for thermocoagulation) weighing between 60 and 90 lbs were obtained from Archer Farms (Darlington, MD, USA) and housed for 1–2 weeks prior to the initiation of experimental procedures. Swine were randomly assigned to a treatment group. At the time of procedure, swine weight and age ranges were 71.6–91.2 lbs and 73–92 days old for the EC-ethanol ablation procedures and 69–90 lbs and 77–95 days old for thermocoagulation procedures.

### 2.2. Devices and Consumables

**Figure 1:**
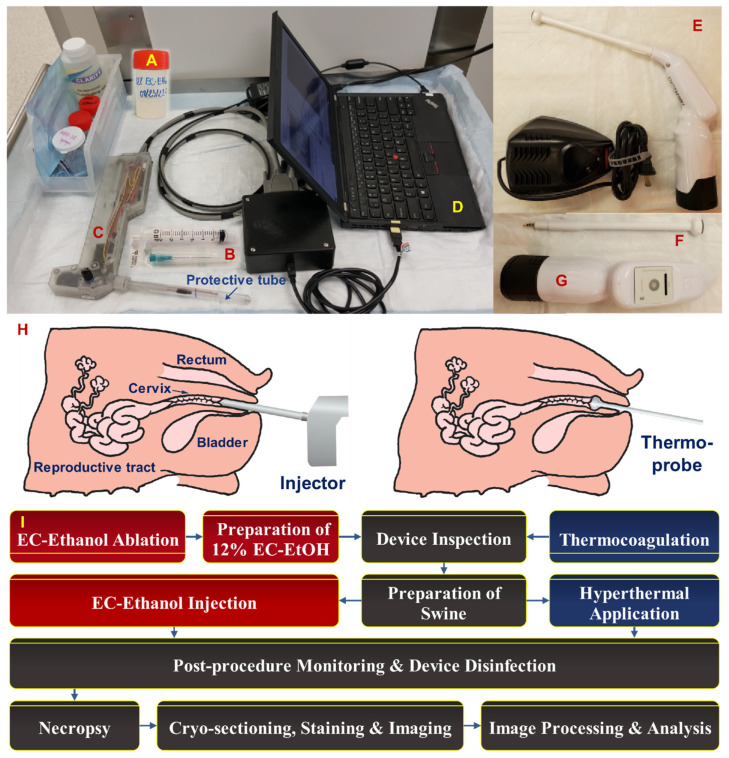
In vivo swine tissue ablation equipment and procedures. Supplies and equipment included: (**A**) a 12% EC-ethanol solution; (**B**) a 23-gauge needle and 3 mL syringe; (**C**) a custom hand-held injector; (**D**) a laptop with a custom interface for controlling injections; (**E**) a Liger Medical thermocoagulator and charger; (**F**) a thermoprobe; and (**G**) a thermooperator. (**H**) Orientation of an injector and thermoprobe within the vaginal canal. (**I**) Overview of study protocol for EC-ethanol ablation and thermocoagulation procedures.

Mixtures of EC (Sigma Aldrich, St. Louis, MO, USA) and ethanol (200 proof—i.e., 100% ethanol, Koptec, King of Prussia, PA, USA) were prepared by mixing at room temperature until all EC was dissolved. Specifically, 12% EC-ethanol solutions (EC to ethanol, weight to weight, i.e., 12% EC to 88% pure ethanol) were prepared less than 24 h prior to EC-ethanol ablation procedures (Figure 1A). EC-ethanol solutions were then loaded into a 3 mL syringe (0.866 mm inner diameter), which was attached via a Luer Lock connection to a custom hand-held injector previously described in [25] (Figure 1B,C). Then, a single-end beveled 23G needle was attached to the tip of the injector. The injector enabled us to locate the cervix and administer the EC-ethanol solution into the swine cervix with controlled injection parameters, including insertion rate and depth, injection volume and rate, and retraction time, which could be controlled via a custom graphical user interface. A built-in camera and LED light source at the probe tip enabled intraprocedural visualization of the cervix injection (Figure 1D). The pressure during each injection was recorded via a built-in manometer.

A commercial thermocoagulator (Liger HTU-110C, Liger Medical LLC, Lehi, UT, USA) shown in Figure 1E–G was used to perform thermocoagulation on the swine cervix with controlled ablation temperature and duration. We selected 100 °C for 20 s because it was within the range of temperatures and heating times used in other studies and was the protocol least likely to cause pain or discomfort [12]. Prior to each use, the thermocoagulator probe was cleaned in two steps, as recommended by the manufacturer: (1) the probe was first soaked in Enzol for 1 min and rinsed with running water, and (2) the probe was subsequently submerged in Cidex (2% glutaraldehyde) for 20 min and then thoroughly rinsed 3 times with de-ionized water.

### 2.3. Protocol for EC-Ethanol Ablation and Thermocoagulation Procedures in Swine Cervix

An overview of the protocol for in vivo EC-ethanol ablation and thermocoagulation in the swine cervix is presented in Figure 1H. After overnight food withdrawal, each pig was sedated via intravenous administration of a Telazol-Ketamine-Xylazine (TKX) cocktail, transferred to the operating room, and secured in the prone position on a gurney with sandbags. Lubricant ophthalmic ointment was applied to prevent eye desiccation, and isoflurane was administered nasally to maintain sedation. Subsequently, either the injector was inserted to inject 500 µL of 12% EC-ethanol into the swine cervix or the thermoprobe was inserted to heat the ectocervix to 100 °C for 20 s to ablate cervical tissue. Vital signs, including body temperature, heart rate, respiratory rate, and blood oxygen (SpO_2_), were measured periodically until the procedure was completed. Once the swine were returned to their housing, post-procedure monitoring of body temperature, heart rate, respiratory rate, and recovery time was conducted. Side effects and adverse events were also documented.

**Figure 2:**
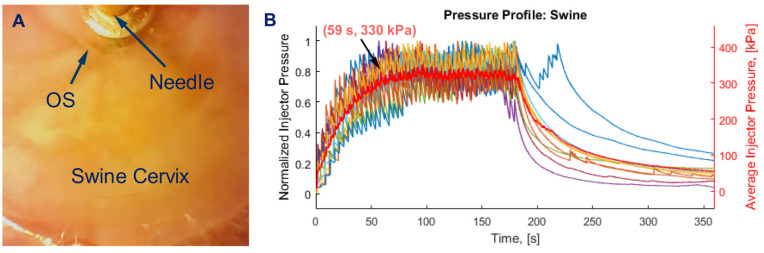
Intraprocedural data during swine cervical ablation procedures. (**A**) Real-time display during EC-ethanol injection. (**B**) Pressure profile during delivery of EC-ethanol into the swine cervix (7.6 mm insertion depth, 10 mm/s insertion rate, 12% EC-ethanol, 10 mL/h infusion rate, 500 μL infusion volume, n = 10 swine). Each colored line denotes one procedure. The red line denotes the average across procedures.

The injection parameters for EC-ethanol were based upon the results of our prior ex vivo study [25]. They included a needle insertion rate of 10 mm/s, an infusion rate of 10 mL/h, a programmed needle insertion depth of 7.6 mm (which achieved a penetration depth of 4.8 mm in tissue [25]—the depth needed to treat high-grade dysplasia), and a retraction time of 300 s. A 23G needle was selected to minimize the pressure build-up within the injector and to stay below the 520 kPa threshold at which the injector would end an injection (pressures higher than 520 kPa could blow apart internal tubing within the injector). The bevel was pointed away from the cervical os to minimize leakage into the endocervical canal. Injections, which were guided by the real-time video, were performed at the 12 o’clock position of the cervix to help maintain consistency across experiments. The injector was tilted upwards at an angle between 10 and 15 degrees relative to the horizon to ensure the needle was adequately inserted into the tissue rather than into the endocervical canal. The built-in camera and manometer in the hand-held injector were used to monitor EC-ethanol injections throughout the procedure. Specifically, the video feed from the camera (illustrated in Figure 2A) was used: (1) to guide needle placement directly above the os, and (2) to monitor for the visual presence of backflow to the tissue surface during the injection. The manometer was used to monitor the pressure during the injection. If the needle was properly inserted into the cervix, the pressure increased and reached an average peak around 330 kPa at 59 s, then stabilized until injections were completed (Figure 2B). Low pressures (<300 kPa) indicated the needle was improperly inserted—either into the endocervical canal or through the cervix into surrounding tissue. High pressures indicated there was a clog in the tubing or needle. The pressure threshold of 520 kPa was set to prevent device damage and leakage away from the injection site.

### 2.4. Processing for Histopathological Assessment

At 24 h post-procedure, swine were euthanized. In n = 1 swine, two EC-ethanol injections were performed 7 days and then 48 h prior to euthanasia in order to initially evaluate the degradation of EC and healing response. All cervices containing the ablation zone were excised, embedded in an optimal cutting temperature (OCT) compound, and flash-frozen in cold 2-methylbutane. Supplementary Figure S1A shows the swine reproductive tracts treated with EC-ethanol ablation (left) and thermocoagulation (right) and the cervical sections excised 24 h post-ablation. The entire necrotic regions caused by EC-ethanol ablation (Supplementary Figure S1B) and thermocoagulation (Supplementary Figure S1C) were identified through palpitation and visual inspection (Section 2). The necrotic tissue has dark/red and white colors. The white and stiff cervical necrosis in Supplementary Figure S1B contained EC deposits. Flash freezing of the cervical sections embedded in OCT with 2-methylbutane is illustrated in Supplementary Figure S1D. After flash freezing, blocks were sectioned at 20 µm thickness with 500 µm spacing between slices and stained with nicotinamide adenine dinucleotide (NADH) diaphorase, which stains viable tissue blue and does not stain necrotic regions. Each section was then imaged using an inverted bright-field microscope (DMi8, Leica Microsystems, Buffalo Grove, IL, USA) at 2.5× magnification.

### 2.5. Semi-Automated Image Processing Algorithms

**Figure 3:**
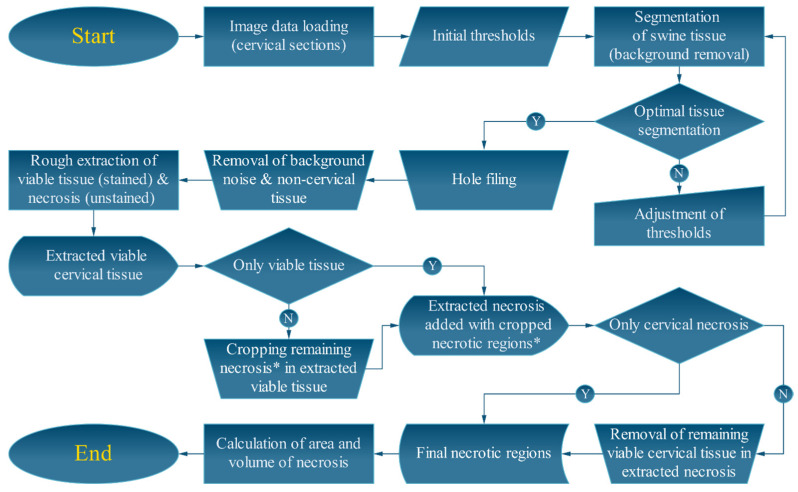
Flowchart depicting semi-automated algorithms for image processing and analysis. (*) denotes that the necrotic regions that remained in the extracted visible cervical tissue were manually segmented and added to the extracted necrosis.

A MATLAB script was developed for semi-automated image processing and analysis of necrosis in cervical sections, as presented in Figure 3. First, the image of each cervical section was loaded into the program, and auto-segmentation of the cervical tissue was performed with initial thresholds. After the background was removed, holes and noise within the segmented regions were selected manually for filling and removal, respectively. Then, necrotic and viable regions were separated for subsequent analysis using color information in each image. If necrosis remained in the viable image or viable tissue remained in the necrosis image, they were manually removed. Subsequently, the viable and necrotic areas in each slice were calculated. Then, the volume of necrosis was calculated as $\left[\sum_{i=1}^{N-1}\left(A^{i}\times 0.52\right)\right]+\left(A^{N}\times 0.02\right)$ and the depth of necrosis as *N* × 0.02 + (*N* − 1) × 0.5, where *A^i^* and *N* are the necrotic area in cervical section *i* and the total number of sections containing cervical necrosis, respectively. Because only the 12 o’clock position in the cervix was injected with EC-ethanol, we also quantified the necrotic volume and depth in the top half of cervices ablated with thermocoagulation; this enabled us to make a more realistic comparison between the two therapies. Specifically, we found the centroid of the cervix and then drew a horizontal line through the centroid and calculated the necrotic area above the line. Then the volume and depth of necrosis were calculated from the necrotic areas in the same way as described above.

### 2.6. Image Alignment and Volume Reconstruction

Cervical sections were not always registered the same on the microscope slides, and thus did not appear over the same ranges of coordinates when imaged under the microscope. We applied rigid transformations (translation and rotation) to align the microscope images. The aligned cervical sections were then loaded into MicroView 3D Image Viewer software version 2.5.0 (Parallax Innovations Inc., Ilderton, ON, Canada) to extract a 3D surface of the necrosis. The necrotic volume was visualized and manipulated in MeshLab (Visual Computing Lab, Pisa, Italy).

### 2.7. Statistical Analysis

All statistical analyses were performed using GraphPad Prism software version 10.0.0 (GraphPad Software, San Diego, CA, USA). Parametric t-tests (unpaired, two-tailed) were performed to compare vital signs, necrotic volumes, and necrotic depths achieved with each treatment. A significance level of *p* = 0.05 was considered to reject the null hypothesis for all analyses.

### 2.8. Data Availability

The data generated in this study are available upon request from the corresponding authors.

## 3. Results

### 3.1. EC-Ethanol Ablation Achieved Comparable Acute Safety Endpoints to That of Thermocoagulation

**Figure 4:**
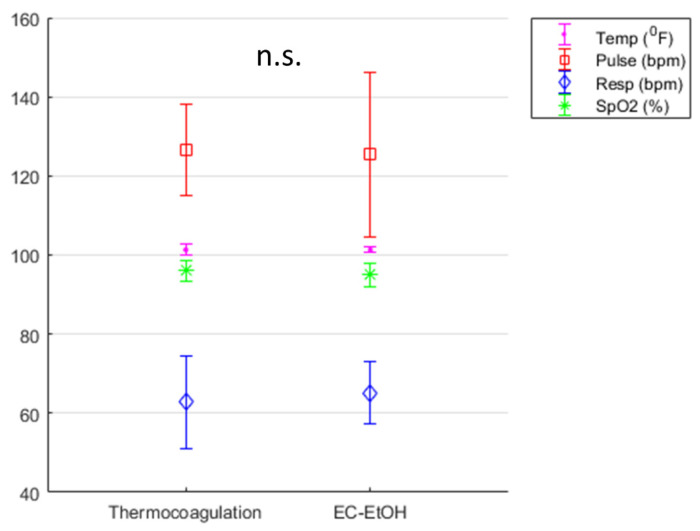
Acute safety data. There were no significant differences between EC-ethanol and thermocoagulation with respect to temperature (temp, 101.3 ± 0.7 °F vs. 101.3 ± 1.4 °F), pulse (125.41 ± 20.74 bpm vs. 126.55 ± 11.6 bpm), respiratory rate (resp, 65.11 ± 7.94 bpm vs. 62.8 ± 11.78 bpm), and oxygen saturation (SpO_2_, 94.94 ± 3.13% vs. 96.1 ± 2.6%), respectively. n.s. = not significant. Error bars = standard deviation.

The vital sign evaluations taken during both procedures were stable. There were no significant differences between EC-ethanol and thermocoagulation with respect to temperature (101.3 °F vs. 101.3 °F), respiratory rate (65.11 bpm vs. 62.8 bpm), and oxygen saturation (SpO_2_, 94.94% vs. 96.1%) (Figure 4). The pulse (heart rate) varied slightly more in the EC-ethanol ablation procedure, but the average (125.41 ± 20.74 bpm) was not significantly different from that of thermocoagulation (126.55 ± 11.6 bpm). All vital signs were within the normal range, as confirmed by a veterinarian. The side effect of vaginal bleeding was observed in 3 pigs treated with thermocoagulation and in 2 pigs treated with EC-ethanol. No adverse events were observed for either treatment group.

### 3.2. EC-Ethanol Ablation Achieved Comparable Depth and Volume of Necrosis in the Upper Half of the Cervix as Compared to That of Thermocoagulation

**Figure 5:**
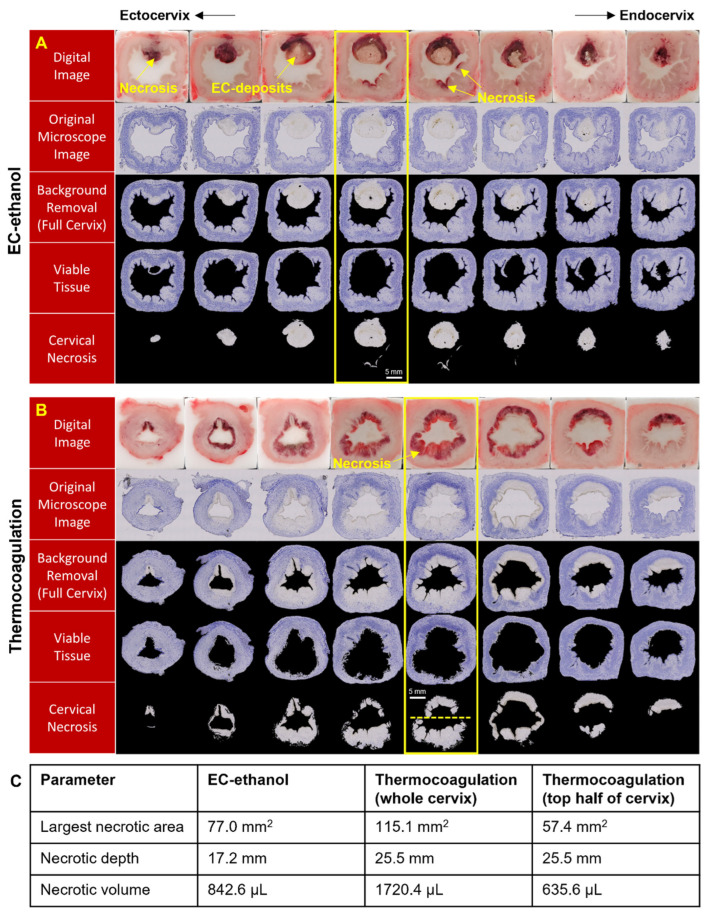
Histological characterization of the ablation zone. Sections of swine cervix treated with (**A**) EC-ethanol ablation (7.6 mm insertion depth, 10 mm/s insertion rate, 12% EC-ethanol, 10 mL/h infusion rate, 500 μL infusion volume, n = 10 swine) and (**B**) thermocoagulation (100 °C for 20 s, n = 10 swine). Necrosis (unstained gray regions) could be clearly seen in the excised cervices. The sections with the maximum necrotic area are outlined in yellow. The dotted yellow line in (**B**) demarcates the top half of the cervix. (**C**) The maximum necrotic area, depth, and volume are 77.0 mm^2^, 17.2 mm, and 842.6 µL for a single injection of EC-ethanol ablation; 115.1 mm^2^, 25.5 mm, and 1720.4 µL for thermocoagulation (whole cervix); and 57.4 mm^2^, 25.5 mm, and 635.6 µL for thermocoagulation (top half of cervix).

The segmentation process used to determine the area of cervical necrosis in each section is illustrated in Figure 5A,B for EC-ethanol ablation and thermocoagulation, respectively. The digital images taken throughout the cryo-sectioning process (in which the necrotic tissue appears dark and the remaining EC deposits appear white) served as references for locating cervical necrosis. In microscopy images, the viable cervical tissues stained with NADH-diaphorase turned blue, while the areas of necrosis and EC deposits were unstained and seen as white or gray [26]. The white background, artifacts, holes, and non-cervical tissue in the original microscopy image were removed to obtain the segmented cervical section displayed on the black background. The necrotic tissue shown in the last row of Figure 5A,B was digitally extracted from the overall ecto-cervix via our semi-automated algorithm. The highlighted cervical sections showed maximum necrotic areas of 77.0 mm^2^ for EC-ethanol ablation, 115.1 mm^2^ for thermocoagulation (whole cervix), and 57.4 mm^2^ for thermocoagulation (top half of cervix) in the representative data shown in Figure 5. The depths and total volumes of the cervical necroses for the representative examples in Figure 5 were 17.2 mm and 842.6 µL for EC-ethanol ablation, 25.5 mm and 1720.4 µL for thermocoagulation (whole cervix), and 25.5 mm and 635.6 µL for thermocoagulation (top half of cervix). The spread of the necrosis induced by EC-ethanol ablation was concentrated around the 12 o’clock position where the ablation was performed and spanned a majority of the upper half of the cervix (i.e., from the endocervical canal to the outer periphery). Conversely, thermocoagulation induced necrosis on both sides of the cervix but primarily treated only the region adjacent to the endocervical canal and not at the periphery.

**Figure 6:**
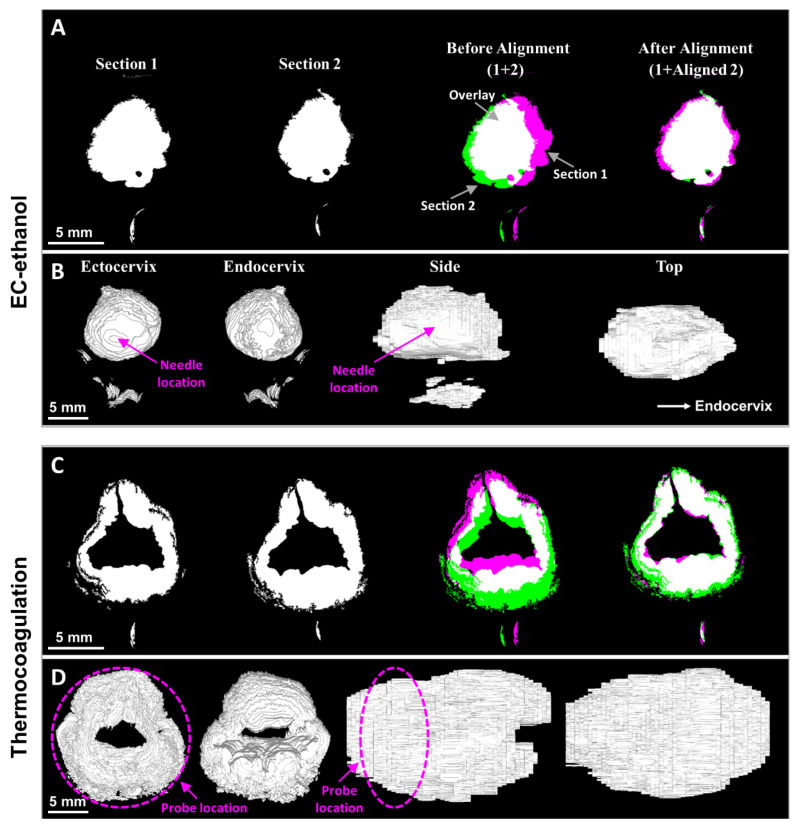
(**A**,**C**) Alignment of cervical sections and (**B**,**D**) 3D rendering of reconstructed necrotic volume for (**A**,**B**) EC-ethanol ablation and (**C**,**D**) thermocoagulation. The 3D reconstructions provide images of the 3D surface of the necrotic volume from various viewpoints, including the ectocervix, endocervix, side view (3 to 9 o’clock slice), and top viewpoint (12 to 6 o’clock slice). Sections 1 and 2 are indicated in pink and green in (**A,C**), respectively. The needle location and probe location are indicated in pink in (**B**,**D**), respectively.

Cervical sections were digitally reconstructed to provide 3D images of the necrotic volumes from EC-ethanol ablation and thermocoagulation (Figure 6). Since the cervical sections (i.e., Sections 1 and 2 in Figure 6A,C) mounted on the microscope slides were not at the same coordinates, 3D rendering of the necroses before image alignment (1 + 2) led to incorrect reconstructions. After Section 2 was transformed to the same coordinates as Section 1, the necrotic volume could be accurately reconstructed. Reconstruction enabled 3D visualization of the shape of the necrosis from the ectocervix, endocervix, side view (3 to 9 o’clock slice), and top viewpoint (12 to 6 o’clock slice) (Figure 6B,D). As seen, EC-ethanol led to a more uniform spread of necrosis (i.e., the ectocervix vs. endocervix and side vs. top views look very similar in their size and shape, Figure 6B). Conversely, thermocoagulation led to more irregular, less predictable spreads of necrosis (i.e., the ectocervix vs. endocervix and side and top views do not look as similar in their size and shape).

**Figure 7:**
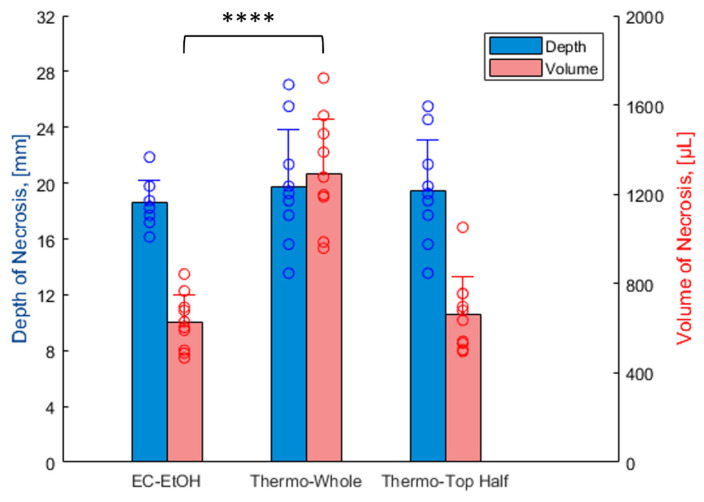
Efficacy measures of a single EC-ethanol injection at the 12 o’clock position (7.6 mm insertion depth, 10 mm/s insertion rate, 12% EC-ethanol, 10 mL/h infusion rate, 500 μL infusion volume, n = 10 swine) compared to thermocoagulation (100 °C for 20 s, n = 10 swine) in the swine cervix model. Average necrotic depth and volume are 18.6 ± 1.6 mm and 626.2 ± 122.8 µL for EC-ethanol ablation, 19.7 ± 4.1 mm and 1292.9 ± 242.3 µL for thermocoagulation (whole cervix), and 19.5 ± 3.7 mm and 664.6 ± 168.5 µL for thermocoagulation (top half of cervix), respectively. **** = *p* < 0.0001. Error bars = standard deviation.

The depth and volume of necrosis observed across all pigs are shown in Figure 7. Comparable depths of necrosis were seen for EC-ethanol (18.6 ± 1.6 mm, with the needle insertion depth set to 7.6 mm) and thermocoagulation (19.7 ± 4.1 mm) (*p* > 0.05). Thermocoagulation produced a significantly larger total necrotic volume of 1292.9 ± 242.3 µL, about twice that of a single 500 µL injection of EC-ethanol at the 12 o’clock position, which produced a necrotic volume of 626.2 ± 122.8 µL (*p* < 0.0001). To enable a more accurate comparison between the two therapies, we also calculated the necrotic volume induced by thermocoagulation in the top half of the cervix (664.6 ± 168.5 µL), which was statistically similar to that of a single EC-ethanol injection (*p* > 0.05). This suggests that two EC-ethanol injections could be performed (e.g., at the 12 and 6 o’clock positions) to achieve comparable volumes to thermocoagulation. Notably, EC-ethanol ablation led to smaller variabilities in necrotic depth and volume compared to thermocoagulation (standard deviations for EC-ethanol are about half that of thermocoagulation), suggesting EC-ethanol ablation may be more precise in inducing repeatable, predictable necrotic zones.

### 3.3. EC Deposits Remain in Cervix for at Least 7 Days and Multiple EC-Ethanol Injections Can Induce Necrosis at Different Positions in the Cervix

**Figure 8:**
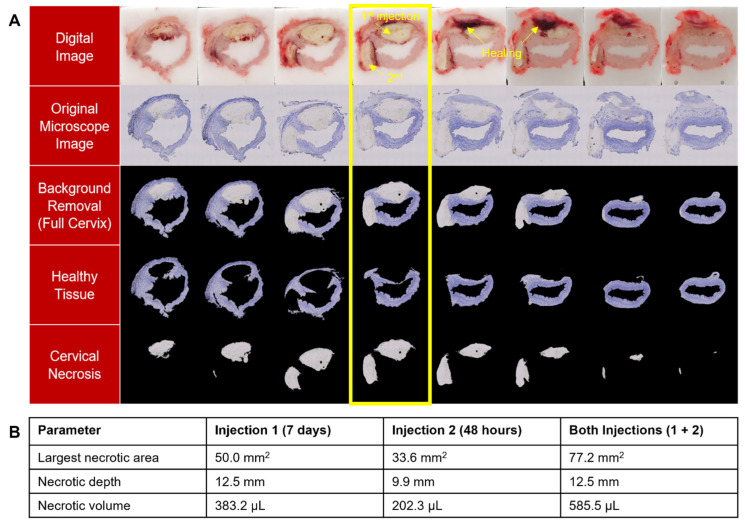
(**A**) Initial time course results with cervix ablated twice: 48 h (9 o’clock position) and 7 days (12 o’clock position) prior to excision (7.6 mm insertion depth, 10 mm/s insertion rate, 12% EC-ethanol, 10 mL/h infusion rate, 500 μL infusion volume for the first and second injections). After 7 days, the EC deposit can still be seen, but the healing process has begun, suggesting EC remains in the cervix for at least 7 days after ablation. The area of necrotic regions from multi-injection of EC-ethanol in the highlighted section is 77.2 mm^2^. The section with the maximum necrotic area is outlined in yellow. (**B**) Total necrotic depth and volume are 12.5 mm and 585.5 µL, respectively.

To initially evaluate how long EC-ethanol remains in the cervix, we performed two 500 µL ablations in n = 1 cervix—one ablation was performed 7 days prior to excision (seen at the 12 o’clock position in Figure 8), and the other was performed 48 h prior to excision (seen at the 9 o’clock position in Figure 8). After 7 days, the EC deposit can still be seen, but the healing process has begun, particularly around the edges of the depot, suggesting EC remains in the cervix for at least 7 days after ablation. This pilot experiment also demonstrated that multiple doses of EC-ethanol can induce necrosis at different positions in the cervix (here 12 o’clock and 9 o’clock). Interestingly, the total necrotic depth (12.5 mm) and volume (585.5 µL) from the two injections excised at 7 days and 48 h after ablation were less than that of a single injection of EC-ethanol (18.6 mm and 626.2 µL) excised 24 h after ablation. This further suggests that there is a healing response occurring in the first 7 days after ablation that is leading to smaller EC deposits over time.

## 4. Discussion

Standard-of-care treatments for cervical dysplasia require specialized equipment and expertise to operate, which limits their implementation in LMICs [3]. EC-ethanol is a potential low-cost treatment option with the potential to overcome limitations associated with current therapies. Specifically, standard injection protocols that achieve predictable zones of necrosis are being developed [25], and treatment depth can be adjusted based on the severity of lesions. Here, for the first time, we thoroughly evaluated if EC-ethanol ablation was able to safely achieve an ablation zone comparable to a clinically accepted therapy for cervical dysplasia, thermocoagulation, in a large animal model. This is a critical step in translating EC-ethanol ablative therapy to women with cervical dysplasia in LMICs.

Vital signs and side effects following EC-ethanol ablation were comparable to those of thermocoagulation, indicating a robust safety profile. All swine undergoing EC-ethanol or thermocoagulation maintained vital signs within normal ranges throughout the procedures (Figure 4). No adverse events were observed for either group. If pure ethanol is injected directly into a small vessel, vascular injury can occur, as documented previously in a case of hepatocellular carcinoma [27]. However, EC likely slows leakage into the vasculature. EC-ethanol has previously been used to treat venous malformations (which are highly vascularized) and was shown to induce sustained local ablation without systemic side effects [28]. Similarly, no systemic side effects were observed in our study. Injection pressure was also monitored with the custom hand-held injector during EC-ethanol ablation since high pressures can: (1) lead to fluid leakage away from the injection site, causing off-target side effects; or (2) destroy tubing within the injector. A consistent pressure profile was maintained during EC-ethanol injections, with average pressure peaks around 330 kPa (Figure 2B), far below the allowed threshold of 520 kPa (beyond which internal tubing within the injector could come apart). In the unlikely event that the pressure reached the maximum allowed value, the probe automatically stopped the injection to prevent any damage to either the instrumentation or the swine.

EC-ethanol and thermocoagulation achieved statistically similar depths of necrosis in our swine study (Figure 7). However, swine anatomy does not contain a true ectocervix, as in humans [24]. In the swine, there is not a significant change in the diameter between the vaginal canal and the endocervical canal. Thus, swine have a much larger diameter endocervical canal that likely propagated heat much further into the swine cervix than it would be in the human cervix; this likely increased the thermocoagulation treatment depth in our study. In this regard, the mean depth of necrosis typically achieved in a single application of thermocoagulation in the human cervix ranges between 2.6 and 3.5 mm [29], vs. 19.7 mm in the swine (Figure 7). While these depths adequately treat low-grade dysplasia, they are not capable of treating all high-grade dysplasia (which can vary in depth from 1.4 to 4.8 mm) or early cancers (i.e., Stage 1A, which can extend 3–5 mm deeper into the stroma) [10,11]. On the other hand, EC-ethanol ablation is likely not impacted by this difference in anatomy, as its induced depth of necrosis is a function of needle depth in the tissue [25]. Here, we selected a shallow needle insertion depth for EC-ethanol to enable comparison to thermocoagulation. Specifically, we programmed the needle insertion depth to 7.6 mm, which, based on previous studies, achieved a penetration depth of 4.8 mm in tissue—the depth needed to treat high-grade dysplasia (the penetration depth is shallower than the programmed needle insertion depth due to tissue deformation or dimpling at the needle tip) [25]. In the future, the needle insertion depth for EC-ethanol ablation will be adjusted depending on the severity of the lesion. This capability could enable EC-ethanol to ablate further than thermocoagulation into the endocervical canal to treat micro-invasive disease or early-stage carcinomas (stage 1A).

Thermocoagulation produced a significantly larger total necrotic volume—about twice that of a single 500 µL injection of EC-ethanol (Figure 7); however, when only the necrosis in the top half of cervices ablated with thermocoagulation was analyzed, EC-ethanol and thermocoagulation achieved statistically similar necrotic volumes. This indicates that two EC-ethanol injections could induce an equal volume of necrosis. EC-ethanol led to a repeatable, predicable necrotic volume (both in shape and spread) that was targeted to one side of the cervix in our experiment (Figure 5, Figure 6 and Figure 7). Such minimizing of collateral damage onto nearby normal cervical tissues while maintaining maximizing local volume distribution around the needle tip is a strength of the gelation process of EC. Additionally, the ability to induce repeatable ablation zones lends itself to multiple injections of EC-ethanol on the same cervix (as demonstrated in Figure 8), which could be guided by low-cost colposcopy previously developed by our group [30,31]. The versatility of EC-ethanol injections to freely and safely select specific regions to ablate has the potential to enable improved cervical dysplasia treatment and management. For example, the different necrotic regions observed from multiple injections, as shown in Figure 8, could help inform needle spacing in the future, leading to standardized protocols for lesion extent and location.

A challenge during our study was determining how to best position the hand-held injector with respect to the cervix prior to performing EC-ethanol ablations. This was further challenged by the biological variability of the swine obtained throughout the study. Due to animal facility constraints, only adolescent pigs under 90 lbs could be housed, resulting in a cohort of swine in different stages of reproductive development. This increased the risk of deploying the EC-ethanol to the wrong location (e.g., beyond the cervix, through the os, into the rectum or urethra), particularly in prepubescent swine, which had smaller, thinner cervices. To mitigate this risk, the pressure was closely monitored during the first minute of the injection to determine if the needle was properly positioned within the cervix, based on the trends observed in previous procedures (Figure 2B). A significantly lower pressure (<300 kPa) than the average indicated improper needle insertion and injection. Specifically, if the pressure did not rise to 300 kPa within the first minute, we observed that the needle had been inserted into the endocervical canal, pierced beyond the cervix, or into the rectum, prompting repositioning of the needle. Another indicator used to identify proper needle placement was the video of the injection, which enabled real-time visualization of EC-ethanol backflow to the tissue surface. If backflow was observed, it indicated a failure to puncture the wall and properly deploy the injectate; this also prompted needle repositioning. Altogether, these correction procedures enabled the identification of robust controls to ensure optimal injector performance and depot formation within the cervix, regardless of the variability of swine development. While these challenges required optimization in swine, the same challenges would likely not be encountered in humans because: (1) speculums are commonly used to gain line of sight to the human cervix (whereas in swine, a camera within the custom injector was required to visualize the cervix); and (2) swine anatomy does not contain a true ectocervix as in the human; thus, the angling of the injector to ensure the needle pierces the cervix would not be required in humans. Rather, a robust, simplified injector that can be used with a standard speculum is being designed by our group for future clinical studies. The injector maintains line of sight through the speculum, thus eliminating the need for a camera and light source and allowing for integration into cervical exam workflows with relative ease.

Our study had several limitations. A majority of swine were euthanized 24 h post-treatment to study the short-term depot formation in the cervical tissue (with the exception of n = 1 swine in Figure 8). Additionally, a single pilot experiment with multiple injections of EC-ethanol was conducted to assess the feasibility and healing response over 7 days. Thus, targets for longitudinal studies in the cervix include assessing long-term safety endpoints, investigating the degradation of the injected EC gel beyond 7 days, evaluating long-term tissue healing of the ablation zone, and determining the ideal spacing of multiple injections. This study used a non-diseased swine cervix to perform injections and evaluate the resulting ablation zones. To the best of our knowledge, no swine models of cervical dysplasia currently exist. To test EC-ethanol in a human-sized diseased model of cervical dysplasia, three-dimensional organotypic culture models could also be used to assess the necrotizing action and degradation of the gel in future studies.

Here, for the first time, we systematically evaluated the safety and efficacy of EC-ethanol injection-induced ablation in a swine cervix model. We found that EC-ethanol ablation had a strong safety profile with no deviations from normal vital signs, limited side effects, and no adverse events within 24 h of ablation. A single 500 µL injection of EC-ethanol ablation induced ablation zones with similar depths and approximately half the total volume compared to thermocoagulation, with the added benefit of enhanced control in selecting needle depth and location(s) within the cervix. This study represents a crucial step towards the translation of EC-ethanol injections for localized tissue ablation, which has the potential to increase women’s access to cervical cancer prevention services in LMICs.

## Data Availability

The data presented in this study are available on request from the corresponding author.

## Supplementary Materials

The following supporting information can be downloaded at: https://www.mdpi.com/article/10.3390/bioengineering10111246/s1, Figure S1: Extraction of treatment zone from harvested reproductive tracts post-ablation. (A) Reproductive tract excised 24 h following EC-ethanol ablation (left) and thermocoagulation (right). Cervical section 2 contains necrotic regions. (B) Necrotic regions caused by EC-ethanol ablation from ectocervix and endocervix viewpoints. (C) Necrotic regions caused by thermocoagulation from ectocervix and endocervix viewpoints. (D) Flash freezing of cervical tissue.
