# Supplementary material for: In Vivo Evaluation of Safety and Efficacy of Ethyl Cellulose-Ethanol Tissue Ablation in a Swine Cervix Model
Source: Bioengineering (Basel). 2023 Oct 25;10(11):1246. doi: 10.3390/bioengineering10111246 (PMC10669649; doi:10.3390/bioengineering10111246)
Supplement: Supplementary file 1 [file bioengineering-10-01246-s001.zip › bioengineering-2639122-supplementary.pdf]

bioengineering

Article

In Vivo Evaluation of Safety and Efficacy of Ethyl Cellulose-Ethanol Tissue Ablation in a Swine Cervix Model

Tri T. Quang [1], Jeffrey Yang [1,2], Michele L. Kaluzienski [1], Anna Parrish [1], Asma Farooqui [1], David Katz [3,4], Brian Crouch [3], Nimmi Ramanujam [3,5,6], and Jenna L. Mueller [1,7,8,*]

1 Fischell Department of Bioengineering, University of Maryland, College Park, MD 20742, USA; tritquang@gmail.com (T.T.Q.); jyang12@umd.edu (J.Y.); mkaluzi4@umd.edu (M.L.K.); annagparrish@gmail.com (A.P.); asmafarooqui101@gmail.com (A.F.)
2 Center for Interventional Oncology, Radiology and Imaging Sciences, NIH Clinical Center, National Cancer Institute, National Institutes of Health, Bethesda, MD 20892, USA
3 Department of Biomedical Engineering, Duke University, Durham, NC 27708, USA; dkatz@duke.edu (D.K.); brian.crouch@duke.edu (B.C.); nimmi.ramanujam@duke.edu (N.R.)
4 Department of Obstetrics and Gynecology, Duke University School of Medicine, Durham, NC 27710, USA
5 Duke Global Health Institute, Duke University, Durham, NC 27710, USA
6 Department of Pharmacology and Cancer Biology, Duke University, Durham, NC 27708, USA
7 Department of OB-GYN & Reproductive Science, University of Maryland School of Medicine, Baltimore, MD 21201, USA
8 Marlene and Stewart Greenebaum Cancer Center, University of Maryland School of Medicine, Baltimore, MD 21201, USA
* Correspondence: mueller7@umd.edu (J.L.M.); Tel.: +1-301-405-8268; Fax: +1-301-405-9953

Supplementary Figure:

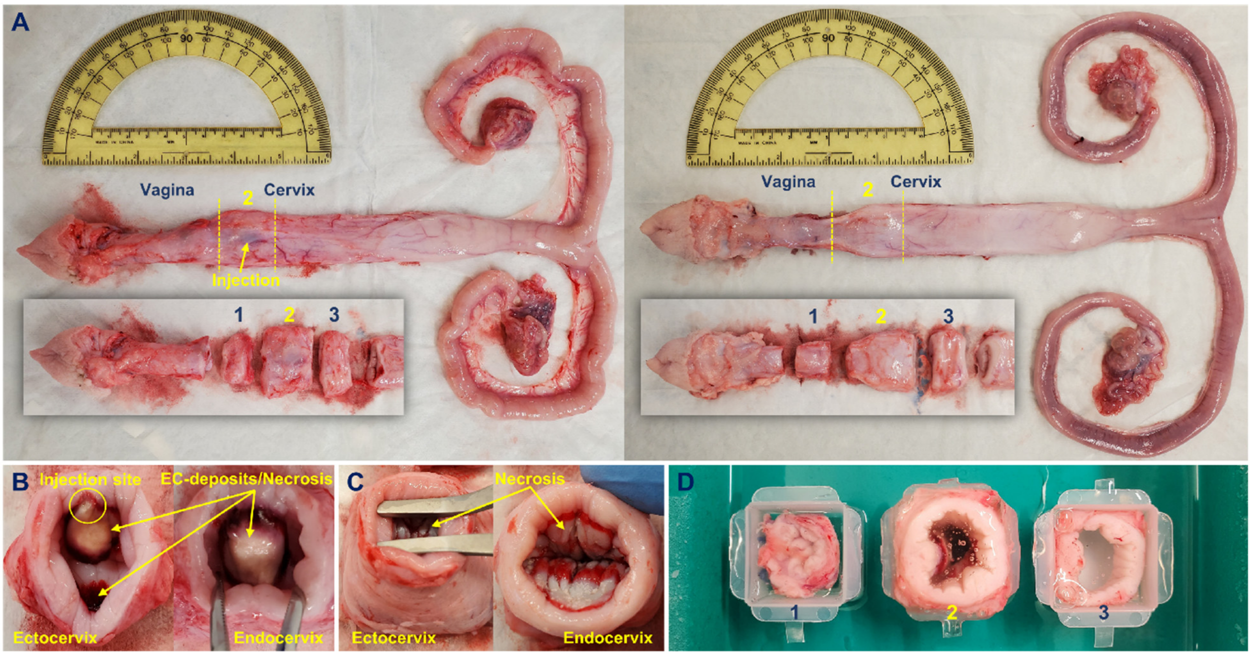

Figure S1. Extraction of treatment zone from harvested reproductive tracts post-ablation. (A) Reproductive tract excised 24 hours following EC-ethanol ablation (left) and thermocoagulation (right). Cervical section 2 contains necrotic regions. (B) Necrotic regions caused by EC-ethanol ablation from ectocervix and endocervix viewpoints. (C) Necrotic regions caused by thermocoagulation from ectocervix and endocervix viewpoints. (D) Flash freezing of cervical tissue.
